# A cell line–derived, immune-competent neurospheroid model to study neuroinflammation and human brain disorders

**DOI:** 10.3389/fimmu.2026.1792896

**Published:** 2026-03-17

**Authors:** Alexandro Angelo Bufi, Andrea Papait, Serafina Farigu, Elsa Vertua, Patrizia Bonassi Signoroni, Elisa Scalvini, Elisabetta Giuzzi, Alice Paini, Paola Chiodelli, Antonietta Rosa Silini, Peter Ponsaerts, Ornella Parolini

**Affiliations:** 1 Department of Life Science and Public Health, Università Cattolica del Sacro Cuore, Rome, Italy; 2 Laboratory of Experimental Hematology, Vaccine and Infectious Disease Institute (Vaxinfectio), University of Antwerp, Antwerp, Belgium; 3 Fondazione Policlinico Universitario “Agostino Gemelli”, Istituto di Ricovero e Cura a Carattere Scientifico (IRCCS), Rome, Italy; 4 Centro di Ricerca E. Menni, Fondazione Poliambulanza Istituto Ospedaliero, Brescia, Italy; 5 Fondazione IRCCS Casa Sollievo della Sofferenza, San Giovanni Rotondo, Foggia, Italy

**Keywords:** 3D model, cell lines, drug screening, hypoxia, microglia, neuroinflammation

## Abstract

**Introduction:**

Three-dimensional (3D) human brain models have become indispensable tools to investigate neuroimmune interactions and inflammatory processes in the human central nervous system *in vitro*. Nevertheless, existing models, including brain organoids and other iPSC-derived systems, are often constrained by lengthy differentiation protocols, considerable cost, and substantial batch-to-batch variability, restricting their applicability in translational neuroimmunology.

**Methods:**

We developed a scalable and reproducible 3D human neurospheroid model (tri-hNSPHs) composed of neuronal, astrocytic, and microglial human cell lines, specifically designed to study neuropathogenic mechanisms. Tri-NSPHs were exposed to a defined pro-inflammatory cytokine cocktail (IL-1β, TNFα, and IFNγ) to quantify the secretion of multiple inflammatory mediators. The inflammation was also counteracted using distinct anti-inflammatory pharmacological compounds and cellular adaptations to hypoxic stress were modeled.

**Results:**

Tri-hNSPHs rapidly self-assemble while maintaining key neuro-glial interactions and enabling precise analysis of immune responses not attainable with conventional two-dimensional cultures. The stimuli we provided triggered robust and quantifiable inflammatory activation, demonstrating the versatility of the model and its suitability for dissecting neuroinflammatory pathways. Pharmacological modulation effectively attenuated these responses, further validating the platform for mechanistic and therapeutic studies. In addition to modeling neuroinflammation, tri-hNSPHs reliably recapitulated cell reactions to hypoxic stress, a pathological condition tightly intertwined to neuroimmune activation in numerous neurological disorders.

**Discussion:**

Together, these findings establish tri-hNSPHs as a scalable, experimentally robust, and translationally relevant 3D neuroimmune model for investigating inflammation-driven brain pathology and evaluating anti-inflammatory strategies in a controlled and reproducible in vitro setting. This platform holds significant promise for advancing neuroimmune research and preclinical screening of immunomodulatory therapies.

## Graphical Abstract

**Figure:**
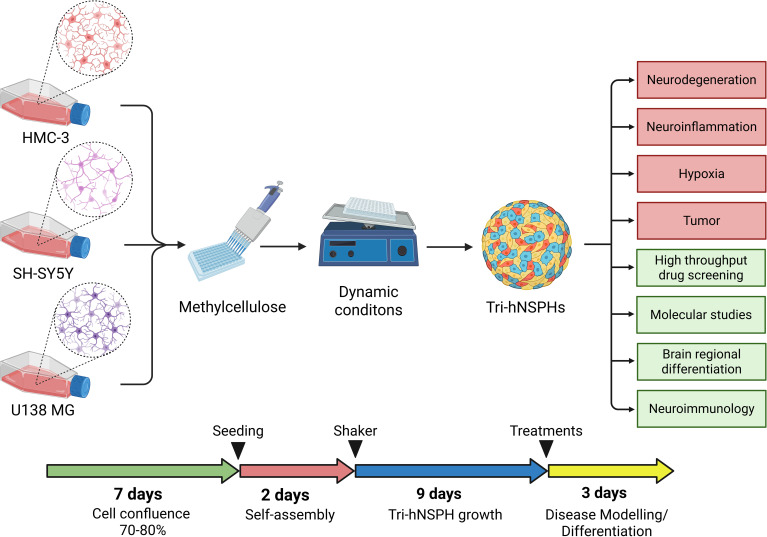

## 1 Introduction

Recapitulating the complexity of the human central nervous system (CNS) with *in vitro* models remains a major challenge, yet a fundamental prerequisite for testing effective therapies for neurological disorders (1–3). A wide range of systems has been employed over the past decades, each with significant limitations. Primary cultures retain intrinsic tissue features, offering a physiologically relevant prototype (4, 5). However, due to the non-proliferative nature of mature neurons, longitudinally spaced experimental designs are hindered (6, 7). Neural stem cells (NSCs) could overcome this issue (8), although their limited localization into specific hippocampus areas makes their isolation technically prohibitive (9). The complex CNS accessibility therefore represents a general challenge for primary neural cultures, confining cell sources to neurosurgical biopsy specimens or post-mortem tissues (10). Immortalized cell lines, while easy to propagate, often carry non-physiological genetic alterations that can obscure disease-relevant phenotypes (11). The introduction of human induced pluripotent stem cells (iPSCs) provided a renewable, patient-specific source of neural cells (12), but their utility in conventional two-dimensional (2D) cultures is limited by the inability to recapitulate the *in vivo* cytoarchitectural organization of cells, crucial for their physiological behavior (13, 14). To address these limitations, three-dimensional (3D) brain organoids derived from iPSCs were introduced (15–17). They consist of cellular aggregates in which the spatial organization enhances both cell–cell and cell–extracellular matrix (ECM) interactions, supporting more physiologically relevant gene expression profiles and cellular functions (16, 18, 19).

Although brain organoids are currently regarded as one of the most advanced and promising platforms for dissecting complex neuropathological mechanisms, they come with inherent limitations (20). The protocols required for their generation are generally lengthy, technically demanding, and quite expensive (21, 22). Batch-to-batch variability poses as a major challenge to reproducibility and restricts the use of organoids for high-throughput or large-scale experimental designs. Therefore, to develop well controlled and reliable differentiation protocols that consistently direct cell commitment toward uniform cellular phenotypes is still something to define (22, 23). Finally, the requirement for specialized equipment and expertise further restricts their widespread adoption in contexts such as drug discovery, genetic screening, and personalized medicine (22).

In light of these challenges, there is a pressing need for experimental platforms specifically designed to investigate neuropathological processes, such as neuroinflammation, in a scalable and reproducible manner, while remaining compatible with high-throughput pharmacological testing. Here, we describe a 3D human neural tissue model, which we refer to as neurospheroids (hNSPHs), engineered to interrogate immune–neural interactions under physiological and pathological conditions. hNSPHs are generated through the rapid self-assembly of human cell lines, enabling the formation of either bipartite (neuronal–astrocytic) or tripartite (neuronal–astrocytic–microglial) configurations. Indeed, this modular design allows the controlled incorporation of microglia, a key immune component of the brain and the main cellular driver of neuroinflammation. The protocol relies on three readily available human cell lines (respectively SH-SY5Y, U138 MG and HMC-3 cells) and the resulting hNSPHs form within two weeks, are highly reproducible, and suitable for high-throughput applications. Importantly, hNSPHs are not intended to recapitulate the full developmental complexity of iPSC-derived organoids, but rather to provide a disease-oriented platform optimized for modeling pathological stimuli relevant to neuroinflammation and brain injury. Accordingly, we demonstrate the responsiveness of hNSPHs to neuroinflammatory and hypoxic challenges, two tightly interconnected processes underlying a wide range of neurological disorders. Finally, we validate the translational potential of this system by testing pharmacological strategies targeting inflammation, using dexamethasone, ibuprofen, and minocycline as proof-of-concept compounds. Collectively, this model represents a pragmatic translational bridge between reductionist 2D cultures and complex organoid systems, specifically tailored for mechanistic studies and therapeutic screening in neuroinflammatory research.

## 2 Materials and methods

### 2.1 Cell culture and differentiation of human neural cell lines

All human cell lines were purchased from American Type Culture Collection (ATCC, Manassas, VA, USA). Neuroblastoma-derived SH-SY5Y cell line (Catalog number: CRL-2266; Lot number: 70055453) was cultured in Eagle’s minimum essential medium (EMEM; Sigma-Aldrich, St. Louis, MO, USA, Catalog number: 56416C) with nutrient mixture Ham’s F12 (EuroClone S.p.A., Pero, MI, Italy, Catalog number: ECB7502L) in 1:1 ratio, supplemented with 10% heat inactivated foetal bovine serum (FBS; Euroclone S.p.A., Catalog number: ECS5000LH), 1% penicillin-streptomycin (P/S; Euroclone S.p.A., Catalog number: ECB3001D), and 1% L-glutamine (Euroclone S.p.A., Catalog number: ECB3000D). Human embryonic microglia HMC-3 cell line (Catalog number: CRL-3304; Lot number: 70054445) was cultured in EMEM with 15% FBS, 1% P/S, and 1% L-glutamine. Glioblastoma-derived U138-MG cell line (Catalog number: HTB-16; Lot number: 70054708) was cultured in EMEM supplemented with 10% FBS, 1% P/S, and 1% L-glutamine.

For the differentiation of SH-SY5Y cells into *neuron-like* cells (NLCs), cells were seeded on Geltrex (Life Technologies, Carlsbad, CA, USA, Catalog number: A1413202) pre-coated plates at a density of 3×10^4^ cells/cm². After 3 days of expansion, cells were treated with 10 μM all trans retinoic acid (ATRA; Sigma-Aldrich, Catalog number: R2625) in Neurobasal medium (NB; Life Technologies; Catalog number: 21103049) with 1X B27 supplement (Life Technologies, Catalog number: 17504-044) and 1% L-glutamine. This treatment included daily medium changes for 5 days. For the differentiation of U138-MG cells into *astrocyte-like* cells (ALCs), they were seeded on Geltrex pre-coated plates at a density of 8×10^3^ cells/cm², allowed to expand for 24 hours, and then cultured for 3 days in medium with 2 mM sodium butyrate (C_4_H_7_NaO_2_ (NaB); Thermo Fisher Scientific, Waltham, MA, USA, Catalog number: A11079).

### 2.2 Immunofluorescence staining

Immunofluorescence staining of 2D cell cultures were performed in µ-Slide 8 Well (Ibidi GmbH, Gräfelfing, Germany, Catalog number: 80826) after 30-minute fixation in 10% neutral buffered formalin (Diapath S.p.A., Martinengo, Italy, Catalog number: F0043). hNSPHs, after 1-hour fixation in 10% neutral buffered formalin, were embedded in optimal cutting temperature (OCT) compound. Ten-µm-thick cryosections were obtained using Leica CM1950 cryostat (Leica Biosystems, Wetzlar, Germany), and collected onto Superfrost Plus (VWR International, Radnor, PA, USA, Catalog number: 631-0108) and Superfrost Plus Gold (VWR International, Catalog number: 630-1324) microscope slides. Fixed cells and slides were washed with PBS (EuroClone, Catalog number: ECB4004LX10) and permeabilized for 1 hour with 0.2% Triton X-100 (Sigma-Aldrich, Catalog number: X100). After washes, a blocking step was performed for 1 hour in normal goat serum (Invitrogen, Thermo Fisher Scientific, Waltham, MA, USA, Catalog number: 1000C). Primary antibodies were incubated overnight at 4°C, and the day after the samples were incubated for 90 minutes at 37°C with the proper combinations of secondary antibodies. Autofluorescence in cryosections was reduced using TrueVIEW Quenching Kit (Vector Laboratories, Newark, CA, USA, Catalog number: 84000), followed by 5min counterstaining with 0.1µg/mL DAPI (Invitrogen, Catalog number: D1306). Slides were mounted with VECTASHIELD (Vector Labs; Catalog number:H-1000-10) and imaged using Leica’s MICA system. Antibody details are in **Table 1**.

**Table 1:** Primary and secondary antibodies employed for immunofluorescence staining.

Primary antibody	Clone	Source	Code	Company	Final concentration
β3-Tubulin	2G10	Mouse	sc-80005	Santa Cruz Biotechnology, Inc., Dallas, TX, USA	2 μg/mL
Nestin	10C2	Mouse	Mab5326	Merck Millipore, Darmstadt, Germany	5 μg/mL
S100B	EP1576Y	Rabbit	AB52642	AbCam plc, Cambridge, UK	9,76 μg/mL
IBA1	Polyclonal	Chicken	PA5-143572	Invitrogen, Thermo Fisher Scientific Inc., Waltham, MA, USA	Dilution: 1:200
MAP2	Ap-20	Mouse	556320	BD Biosciences (San Jose, CA, USA)	2,5 μg/mL
Synapsin	A-8	Mouse	sc-376623	Santa Cruz Biotechnology, Inc., Dallas, TX, USA	4 μg/mL
HLA-DR	TAL 1B5	Mouse	sc-53319	Santa Cruz Biotechnology, Inc., Dallas, TX, USA	4 μg/mL
HIF-1α	54/HIF-1α	Mouse	610959	BD Biosciences (San Jose, CA, USA)	2,5 μg/mL
Secondary antibody	Fluorescence	Source	Code	Company	Final concentration
Anti-mouse	CY5	Horse	CY-2500	Vector Laboratories, Inc., Burlingame, CA, USA	10 μg/mL
Anti-mouse	DyLight-488	Horse	DI-2488	Vector Laboratories, Inc., Burlingame, CA, USA	10 μg/mL
Anti-rabbit	DyLight-488	Goat	DI-1488	Vector Laboratories, Inc., Burlingame, CA, USA	10 μg/mL
Anti-rabbit	DyLight-594	Goat	DI-1594	Vector Laboratories, Inc., Burlingame, CA, USA	10 μg/mL
Anti-chicken	AlexaFluor-568	Goat	A11041	Vector Laboratories, Inc., Burlingame, CA, USA	5 μg/mL

### 2.3 Cell proliferation assay

Cell proliferation was assessed by 5-ethynyl-2′deoxyuridine (EdU) incorporation. First, 10 µM EdU was added to U138-MG cells after 3 days of differentiation. After 16–18 hours, cells were harvested and stained with fixable viability dye EF780 (Life Technologies, Catalog number: 65-0865-14) for the exclusion of dead cells. Subsequently, samples were fixed in 0.5% formaldehyde methanol-free (Life Technologies, Catalog number: 28906), then EdU incorporation was evaluated by adding 2.5 mM Azide-fluor 488 in the buffer solution containing 100 mM Tris-HCl pH 8.0, 10 mM L-ascorbic acid and 2 mM (CuSO_4_) at room temperature for 30 minutes. Samples were acquired using FACSymphony A3 with FACSDiva Software v8.0 (BD Biosciences, San Jose, CA, USA), and the percentage of proliferating EdU-positive cells quantified with FlowJo v10.8.0 (FlowJo LLC, Ashland, OR, USA). The procedure and reagents are pulled out from Click-iT EdU Alexa Fluor 488 flow cytometry assay kit (Thermo Fisher Scientific, Catalog number: C10420).

### 2.4 Generation of 3D human NSPH models

To generate the human bipartite neurospheroid model (bi-hNSPHs), SH-SY5Y and U-138 MG cells were harvested upon reaching approximately 70% confluence and combined into a single cell suspension mixed at a ratio of 1:1. For the human tripartite neurospheroid model (tri-hNSPHs), which included microglial cells, SH-SY5Y, U-138 MG, and HMC3 cells were seeded at a defined ratio of 1:2:4, respectively. Each hNSPH model had a final density of 1.2×10^4^ cells/well. Cells were plated in untreated U-bottom 96-well plates (Merck Life Science S.r.l., Milan, MI, Italy, Catalog number: CLS3788) in NB with 1X B27 and supplemented with 0.4% methylcellulose (Merck Life Science S.r.l., Catalog number: M7027), in a total volume of 200µL/well. hNSPHs were cultured for 14 days, with half-medium changes performed every other day, and in dynamic condition using a rocking platform.

### 2.5 Haematoxylin and eosin staining

For H&E staining, bi- and tri-NPSH cryosections were immersed in 95% ethanol (Diapath S.p.A., Martinengo, BS, Italy, Catalog number: A0166) and then in double distilled water (ddH_2_O), followed by incubation in Carazzi’s hematoxylin (Diapath S.p.A., Catalog number: C0203) for 4 minutes. After rinsing under running tap water, counterstaining was carried out for 2 minutes using eosin Y (Diapath S.p.A., Catalog number: C0353). Slides were subsequently dehydrated in 95% and then 100% ethanol and finally cleared in xylene prior to mounting with Eukitt (Sigma-Aldrich, Catalog number: 25608-33-7).

### 2.6 Live&Dead staining

Upon reaching complete maturation, tri-hNSPHs were pipetted singularly in µ-Slide 8 Well, washed with PBS to clear phenol-red and incubated 1 hour in BrainPhys imaging optimized neuronal medium (StemCell Technologies Inc., Vancouver, BC, Canada; Catalog number: 05796). Tri-hNSPHs were then incubated for 45 minutes with 2.5 µM Calcein-AM (Sigma-Aldrich, Catalog number: 17783) and 2.5 µg/mL Hoechst 33342 (Invitrogen, Catalog number: H1399) on a rocking platform to allow in-depth labeling. Before imaging, tri-hNSPHs were counterstained with 2 µg/mL propidium iodide (Sigma-Aldrich, Catalog number: P4864) for 10 minutes and washed twice before imaging.

### 2.7 Pro- and anti- inflammatory stimulations

In order to trigger inflammation, the cocktail of administered cytokines comprised recombinant human (rh) IFN-γ (50ng/mL; BD Biosciences; Catalog number:554616), rhTNF-α (5ng/mL; Miltenyi Biotec; Catalog number: 130-094-014), and rhIL-1β (10ng/mL; Miltenyi Biotec; Catalog number:130-093-895). Dexamethasone (1 µM; Merck Life Science S.r.l.; Catalog number: D4902), Ibuprofen (400 µM; Merck Life Science S.r.l.; Catalog number: I4883) and Minocycline (5 µM; Merck Life Science S.r.l.; Catalog number: M9511) were tested as anti-inflammatory drugs, so they were singularly administered to tri-hNSPHs, either untreated or stimulated with the pro-inflammatory cocktail. After 3days of treatment, tri-hNSPHs were starved for 24 hours in NB without supplements to collect supernatants.

### 2.8 Inflammatory mediator quantification: multiplex bead-based immunoassay and ELISA

Cytokine concentrations were quantified in supernatants collected from tri-hNSPHs stimulated as previously described. Twenty-four hours before supernatant collection, medium containing the treatments were removed and tri-hNSPHs were incubated in NB supplemented with 1% L-glutamine and 1% P/S to enable the secretion of factors stimulated under each treatment condition. Supernatants from hypoxic tri-hNSPHs were collected without starvation to keep the effects caused by oxygen deprivation. After harvesting the supernatants, they were centrifuged to remove cell components, aliquoted and stored at −80°C. Supernatants from unstimulated tri-hNSPHs were included as controls. Each sample was thawed immediately before analysis. Cytokine levels were measured using a multiplex bead-based immunoassay (BD CBA Flex Set, BD Biosciences). Assays were conducted in accordance with the manufacturer’s instructions. Data acquisition was performed using a FACSymphony A3 with the FACSDiva Software v8.0 (BD Biosciences), and analysis was carried out using FCAP Array™ software 3.0 (BD Biosciences). PGE_2_ was quantified with the Human Prostaglandin E_2_ ELISA kit (Invitrogen, Catalog number: KHL1701) following the instructions of the provider. Absorbance was measured at 405 nm using the Victor X4 plate reader (PerkinElmer, Waltham, MA, USA). The cytokines that were measured are listed in **Table 2**.

**Table 2:** Cytokine analyzed with multiplex bead-based immunoassay.

Cytokine	Bead position	Catalog number
IL-6	A7	558276
IL-8	A9	558277
IL-1β	B4	558279
CXCL10	B5	558280
CCL2	D8	558287
CCL5	D4	558324
VEGF	B8	558336

### 2.9 Hypoxia

After 14 days of maturation, hypoxia was chemically mimicked in tri-hNSPHs incubating them with 200 µM cobalt chloride (CoCl_2_; Merck Life Science S.r.l., Catalog number: C8661) for 24 hours on the rocking platform. Hypoxia was also induced cultivating the tri-hNSPHs inside an incubator with 1% oxygen for 24 hours.

### 2.10 Western blot

The nuclear protein extraction for the tri-hNSPHs treated for the induction of hypoxia was performed using the Nuclear Extraction Kit (Novus Biologicals, LLC, Centennial, CO, USA, Catalog number: NBP2-29447) according to the manufacturer’s instructions. To obtain complete lysis of the tri-hNSPHs, after 1 hour incubation in Nuclear Lysis Buffer the samples were subjected to 5 cycles of 15-second-long sonication. To concentrate the total protein content, the nuclear fraction was collected in 25 µl that were entirely loaded onto a precast 4-15% SDS-PAGE gel (BD Biosciences, Catalog number: 4561083). Samples were analyzed by Western blot using the primary antibody targeting HIF-1α (BD Biosciences, Catalog number: 610959) and probed with the anti-mouse-HRP secondary antibody (Bio-Rad; Catalog number: 170-6516) and incubated for 90 minutes at room temperature. Therefore, for the normalization, membranes were stripped for 15 minutes with StripAblot (EuroClone S.p.A., Catalog number: EMP100500) and the primary antibody against GAPDH (Bio-Rad; Hercules, CA, USA; Catalog number: MCA4740) was incubated overnight. Lastly, the membranes were probed with the same secondary antibody. Images were acquired with iBright 1500 Imaging System (Thermo Fisher Scientific) and were analyzed using the iBright Analysis Software (Thermo Fisher Scientific) that allows band detection, densitometry and normalization workflows.

### 2.11 Molecular and signaling pathway analysis with qRT-PCR

For gene expression analysis, cells and tri-hNSPHs were lysed in RLT buffer, and samples were stored at -80 °C. Total RNA was extracted using the EZ1 RNA Cell Mini Kit (Qiagen, Frederick, MD, USA, Catalog number: 959034) on the BioRobot EZ1 Advanced XL Workstation. cDNA synthesis was performed using the iScript Advanced cDNA Synthesis Kit (Bio-Rad Laboratories, Hercules, CA, USA, Catalog number:1725038). The resulting cDNA was pre-amplified with SsoAdvanced PreAmp Super-mix (Bio-Rad Laboratories, Catalog number: 1725160) before the qRT-PCR run with Sso Advanced Universal SYBR Green Supermix (Bio-Rad Laboratories, Catalog number: 1725274) on Bio-Rad CFX96 Quantitative Real-Time PCR. Data were processed using the Bio-Rad CFX Maestro 2.2 software (Bio-Rad Laboratories). Genes and forward/reverse sequences of the primers used are provided in **Table 3**.

**Table 3:** Genes and forward/reverse sequences of primers used for qRT-PCR.

Gene	Primer forward	Primer reverse
C3	GTGGAAATCCGAGCCGTTCTCT	GATGGTTACGGTCTGCTGGTGA
CCL20	GCTGCTTTGATGTCAGTGCT	GCAGTCAAAGTTGCTTGCTG
COX2	CGGTGAAACTCTGGCTAGACAG	GCAAACCGTAGATGCTCAGGGA
CXCL1	CATCGAAAAGATGCTGAACAGT	ATAAGGGCAGGGCCTCCT
CXCL2	GGCAGAAAGCTTGTCTCAACCC	CTCCTTCAGGAACAGCCACCAA
Gbp2	GTTCCTACATCCTCAGCCATTCC	CCACTGCTGATGGCATTGACGT
GLUT1	CCTGCAGTTTGGCTACAACAC	CAGGATGCTCTCCCCATAGC
GM-CSF	ATGATGGCCAGCCACTACAA	CTGGCTCCCAGCAGTCAAAG
Hmox1	CCAGGCAGAGAATGCTGAGTTC	AAGACTGGGCTCTCCTTGTTGC
IL-12α	TGCCTTCACCACTCCCAAAACC	CAATCTCTTCAGAAGTGCAAGGG
IL-8	GAGAGTGATTGAGAGTGGACCAC	CACAACCCTCTGCACCCAGTTT
iNOS	GCTCTACACCTCCAATGTGACC	CTGCCGAGATTTGAGCCTCATG
LCN2	GTGAGCACCAACTACAACCAGC	GTTCCGAAGTCAGCTCCTTGGT
LDHA	AACATGGCAGCCTTTTCCTT	TAAGACGGCTTTCTCCCTCT
MAFG	AGGAGATCGTCCAGCTGAAGCA	TCTGCTTCTCCAGCTCCTCCTT
Mash1	TCTCATCCTACTCGTCGGACGA	CTGCTTCCAAAGTCCATTCGCAC
MMP3	CTCACAGACCTGACTCGGTT	CACGCCTGAAGGAAGAGATG
NeuroD1	GGTGCCTTGCTATTCTAAGACGC	GCAAAGCGTCTGAACGAAGGAG
NF-κB	GCAGCACTACTTCTTGACCACC	TCTGCTCCTGAGCATTGACGTC
NGF	ACCCGCAACATTACTGTGGACC	GACCTCGAAGTCCAGATCCTGA
NLRP3	GATCTTCGCTGCGATCAACA	GGGATTCGAAACACGTGCATTA
Nurr1	AAACTGCCCAGTGGACAAGCGT	GCTCTTCGGTTTCGAGGGCAAA
PDK1	CTGGGTAATGAGGATTTGACTGT	AAGTCTGTCAATTTTCCTCAAAGG
RPLP0	TGGTCATCCAGCAGGTGTTCGA	ACAGACACTGGCAACATTGCGG
RPS18	GCAGAATCCACGCCAGTACAAG	GCTTGTTGTCCAGACCATTGGC
SERPING-1	GCATCAAAGTGACGACCAGCCA	GTCTCTGTCAGTTCCAGCACTG
STAT3	GGCCCCTCGTCATCAAGA	TTTGACCAGCAACCTGACTTTAGT
VEGFA	TTGCCTTGCTGCTCTACCTCCA	GATGGCAGTAGCTGCGCTGATA
YWHAZ	ACCGTTACTTGGCTGAGGTTGC	CCCAGTCTGATAGGATGTGTTGG
β-Actin	GGATGCAGAAGGAGATCACTG	CGATCCACACGGAGTACTTG

### 2.12 Image data and statistical analysis

**Figure 1:**
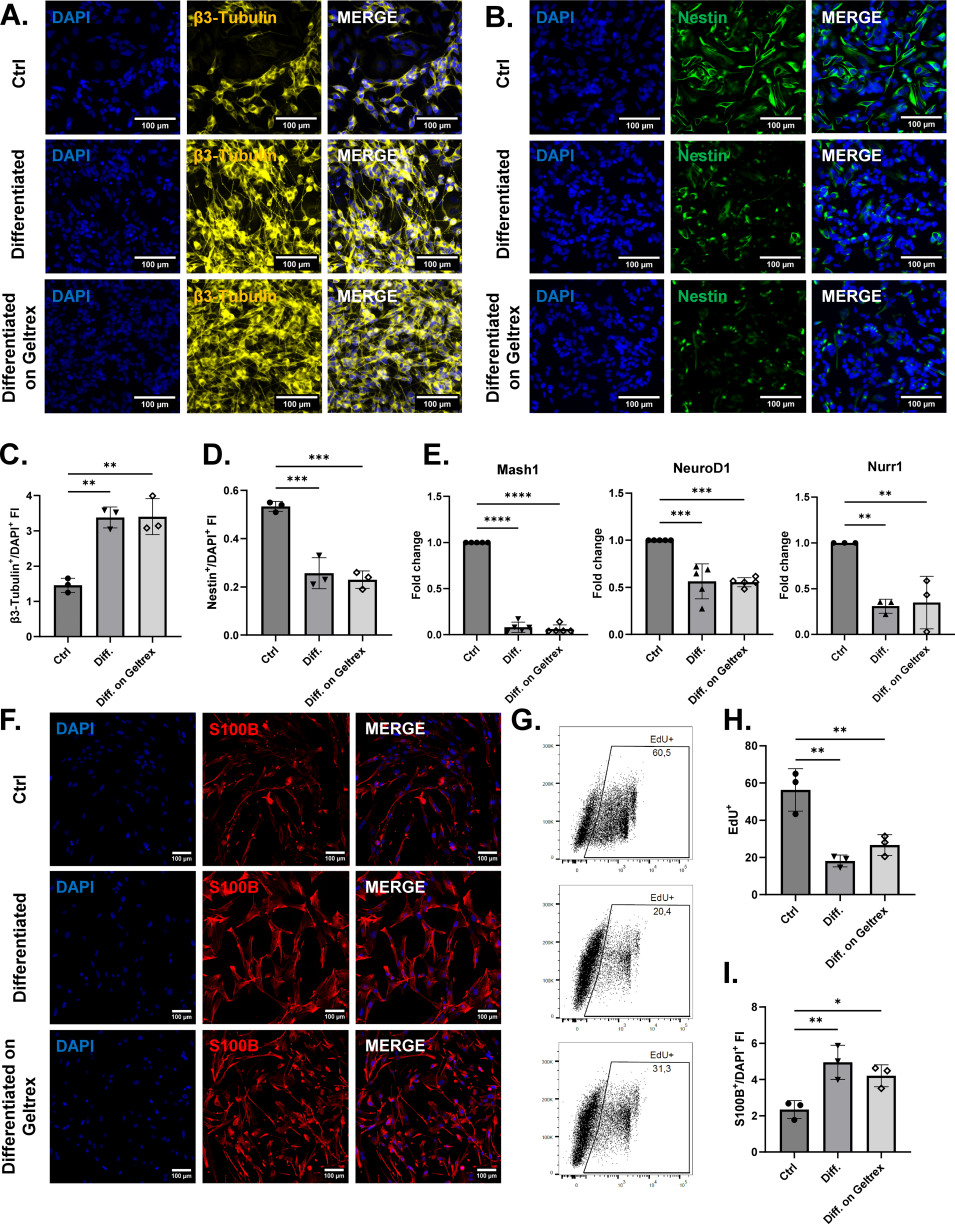
SH-SY5Y and U138 MG cell differentiation into neuron-like (NLCs) and astrocyte-like cells (ALCs). **(A, B)** Representative immunofluorescence images of β3-tubulin (in yellow) and Nestin (in green) for undifferentiated SH-SY5Y cells (Ctrl) and NLCs differentiated on plastic (Diff.) or on Geltrex (Diff. on Geltrex). Nuclei were counterstained with DAPI (blue). **(C, D)** Quantification of the fluorescence intensity for β3-tubulin and Nestin normalized to DAPI signals. **(E)** Relative mRNA expression levels of the proneural transcription factors *Mash1*, *Nurr1*, and *NeuroD1* in undifferentiated and differentiated SH-SY5Y cells. **(F)** Representative S100B (in red) immunofluorescence images for undifferentiated U138 MG cells or ALCs differentiated on plastic (Diff.) or on Geltrex (Diff. on Geltrex). **(G, H)** Quantification of the U138 MG proliferating fractions in differentiated and control conditions measuring the EdU-incorporating cells. **(I)** Quantification of the S100B fluorescence intensity normalized to DAPI signals. Data are shown as mean ± SD from a minimum of three independent replicates (n ≥ 3). Statistical significance was determined by one-way ANOVA followed by Tukey’s *post hoc* test. *p< 0.03; **p< 0.02; ***p< 0.0002; ****p< 0.0001. Scale bar: 100μm. FI, Fluorescence intensity; Mash1, Mammalian achaete-scute homolog 1; NeuroD1, Neuronal differentiation 1; Nurr1, Nuclear receptor related 1 protein.

**Figure 2:**
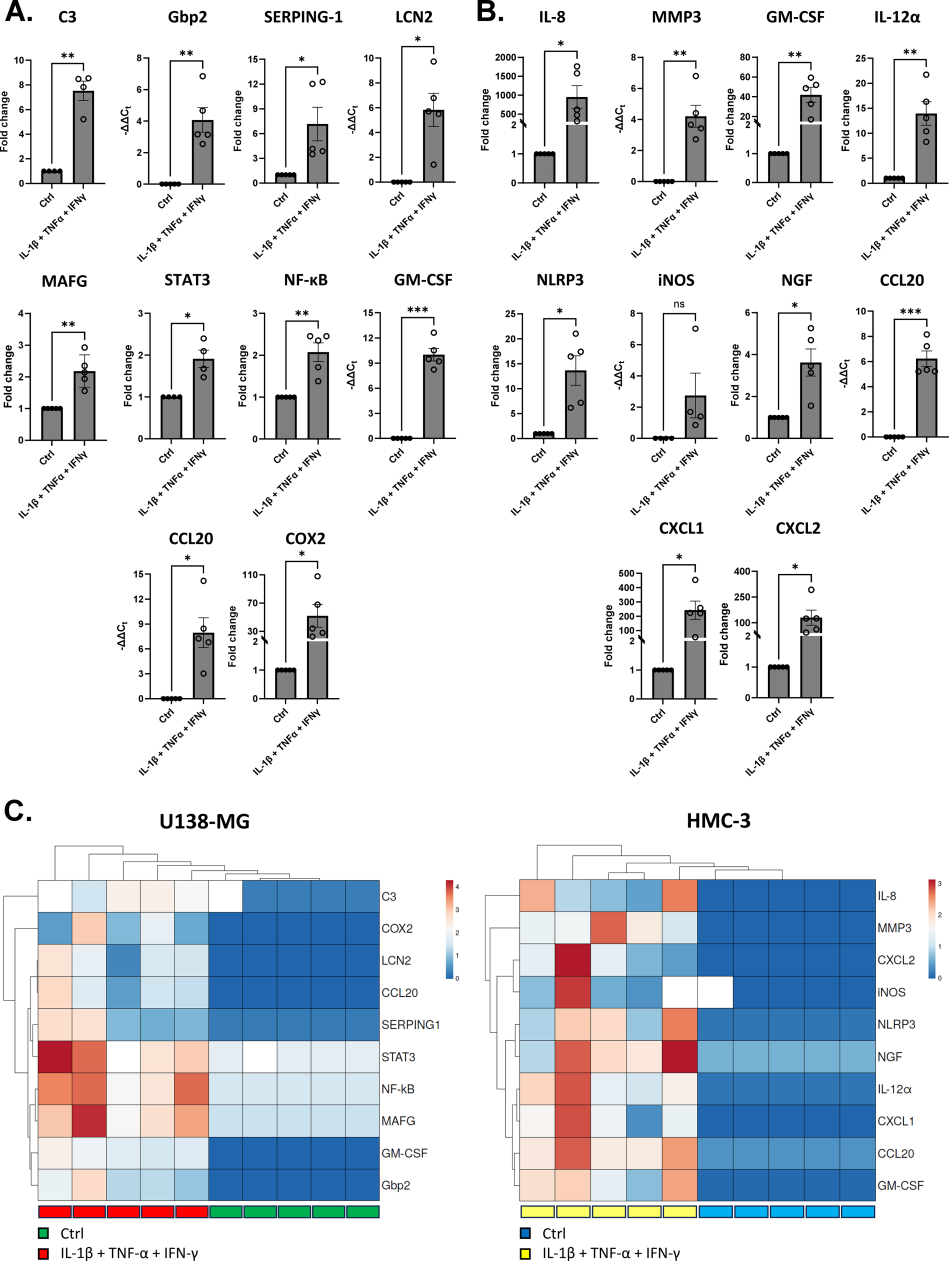
Reactive transcriptional shift of ALCs and HMC-3 cells after pro-inflammatory stimulation. **(A)** Relative mRNA expression levels of pro-inflammatory astrocytic markers, reactive transcription factors and inflammatory mediators in ALCs following cytokine exposure. **(B)** Relative mRNA expression levels of microglial activation–associated genes in HMC-3 cells under the same stimulation conditions. All analyzed genes showed a statistically significant increase in expression upon stimulation, except for iNOS. Data are shown as fold change mean ± SEM from 5 independent replicates (n = 5). Statistical significance was determined by two-tailed unpaired t-test with Welch’s correction. Outliers were identified with ROUT method (Q = 1%) and excluded from the analysis *p< 0.03; **p< 0.02; ***p< 0.0002. Gbp2, Guanylate-binding protein 2; SERPING-1, Serpin family G member 1; LCN2, Lipocalin 2; MAFG, MAF BZIP Transcription Factor; STAT3, Signal transducer and activator of transcription 3, NF-κB, Nuclear factor kappa-light-chain-enhancer of activated B cells, GM-CSF, Granulocyte-macrophage colony stimulating factor; CCL20, C-C motif chemokine ligand 20; COX2, Cyclooxygenase-2, IL-8, Interleukin-8; MMP3, Metalloproteinase 3, IL-12α, Interleukin 12-alpha; NLRP3, NLR family, pyrin domain-containing 3; iNOS, Inducible nitric oxide synthase; NGF, Nerve growth factor; CXCL1, C-X-C motif chemokine ligand 1; CXCL2, C-X-C motif chemokine ligand 2.

**Figure 3:**
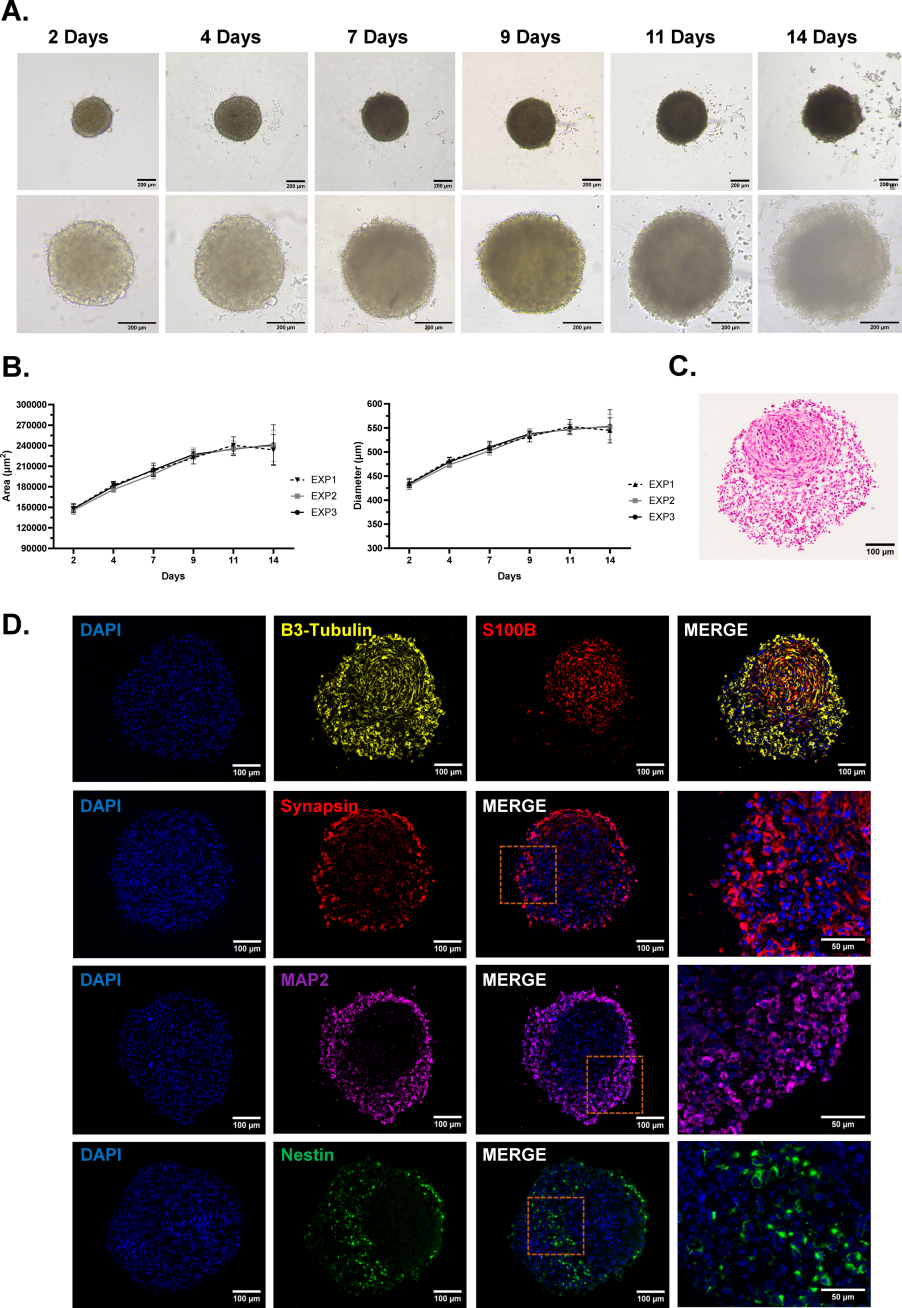
Formation and morphological assessment of bi-hNSPHs. **(A)** Representative bright-field microscopy images showing the formation and growth of bi-hNSPHs within 14 days. Scale bar: 200 µm. **(B)** Growth curves based on the measurement of bi-hNSPHs areas (µm²) and diameters (µm) every other day. **(C)** H&E staining of bi-hNSPHs cryosection at 14 days to reveal their internal cellular disposition. Scale bar: 100 µm. **(C)** Representative immunofluorescence images of neuronal markers for SH-SY5Y – β3-tubulin in yellow, Synapsin in red, MAP2 in magenta and Nestin in green – and astrocytic markers for U138 MG – S100B in red. **(D)** MAP2, Microtubule associated protein 2.

**Figure 4:**
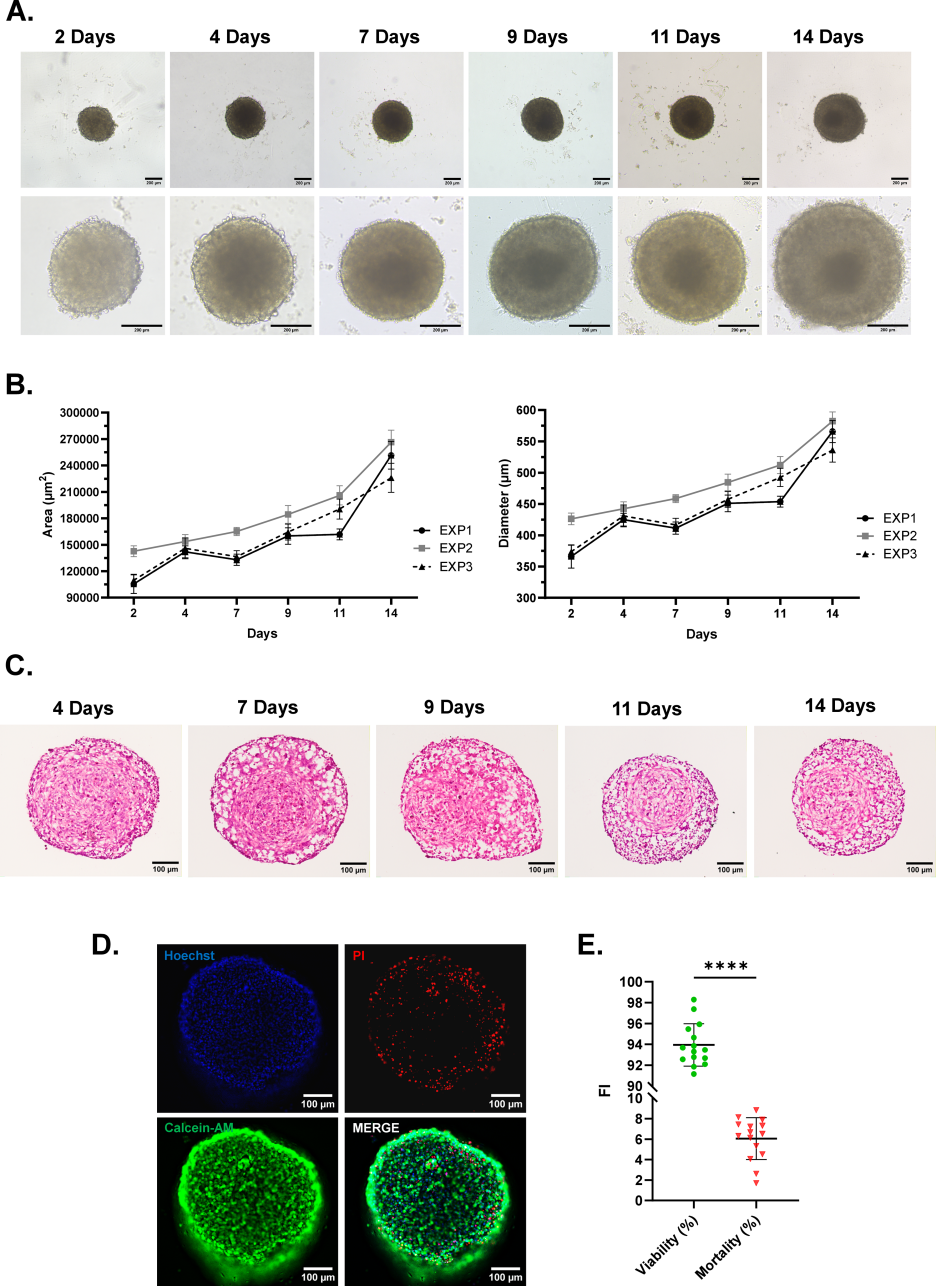
Generation and morphometrical evaluation of tri-hNSPHs. **(A)** Representative bright-field microscopy images showing the maturation of tri-hNSPHs over 14 days. Scale bar: 200 µm. **(B)** Growth curves of tri-hNSPHs in terms of area (µm²) and diameter (µm) over 14 maturation days. **(C)** H&E staining of tri-hNSPH cryosections at different time points to show their internal cellular architecture refining during time. Scale bar: 100 µm. **(D)** Representative fluorescence images of the surface of a tri-hNSPH stained for calcein (live cells, in green) and PI (dead or stressed cells, in red). Scale bar: 100 µm. **(E)** Relative quantification of fluorescence intensity from live and dead cells expressed as viability and mortality percentages. For D, statistical significance was determined with paired t-test and data are shown as mean ± SD from 15 different tri-hNSPHs (n=15). ****p< 0.0001. PI, Propidium iodide; FI, Fluorescence intensity.

**Figure 5:**
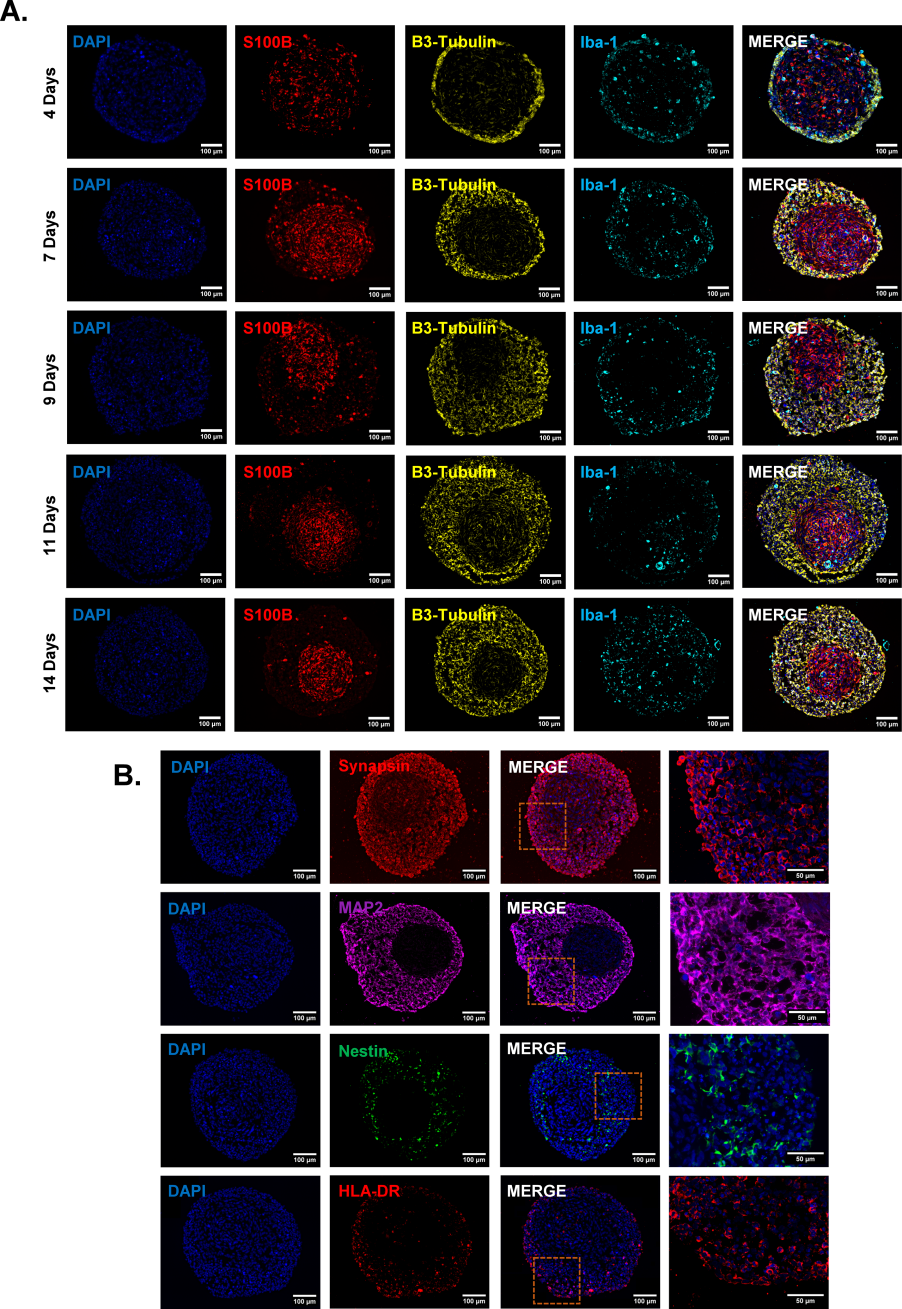
Internal composition of tri-hNSPHs. **(A)** Representative immunofluorescence images of tri-hNSPH cryosections at 4, 7, 9, 11, and 14 days of culture. Sections were stained for the astrocyte marker S100B (in red), the neuronal marker β3-tubulin (in yellow), and the microglia/macrophage marker Iba-1 (in cyan). Nuclei were counterstained with DAPI (blue). **(B)** Representative immuno-fluorescence images of neuronal – Synapsin in red, MAP2 in magenta and Nestin in green – and microglial – HLA-DR in red – markers on cryosectioned tri-hNSPHs. Scale bar: 50-100 µm. Iba-1, Ionized calcium-binding adapter molecule 1; MAP2, Microtubule associated protein 2.

**Figure 6:**
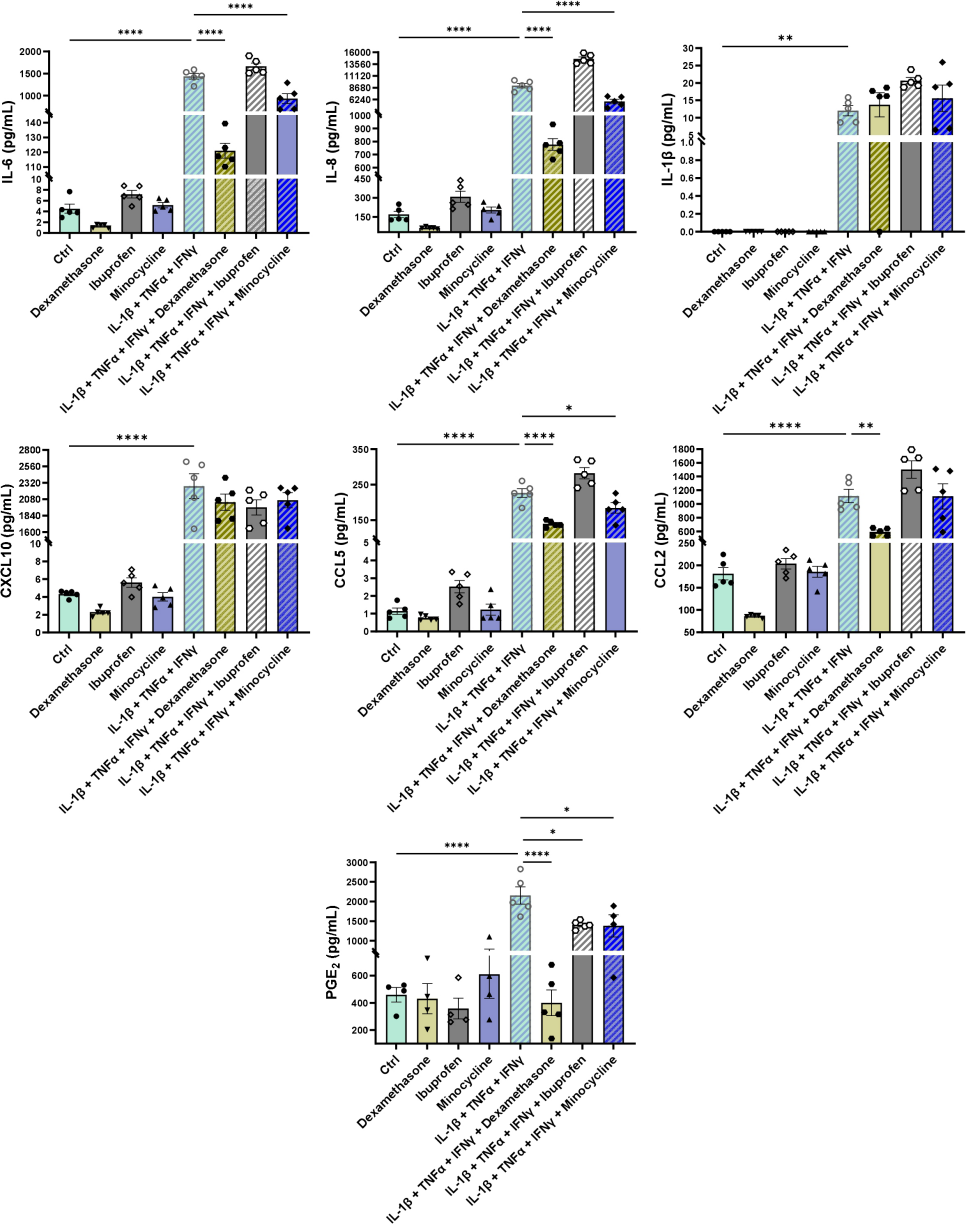
Inflammatory induction and anti-inflammatory treatments of tri-hNSPHs. Quantification of inflammatory mediators in supernatants collected form tri-hNSPHs after three days of treatment with dexamethasone, ibuprofen and minocycline alone; with the pro-inflammatory mix of cytokines (IL-1β, TNF-α and IFN-γ) and with the pro-inflammatory cocktail combined with the three anti-inflammatory compounds. Data are shown as mean ± SD from 5 independent replicates (n = 5). Statistical significance was determined by one-way ANOVA followed by Tukey’s *post hoc* test. *p< 0.03; **p< 0.02; ****p< 0.0001. IL-6, Interleukin-6, IL-8, Interleukin-8; CXCL10, C-X-C motif chemokine ligand 10; CCL2, C-C motif chemokine ligand 2; CCL5, C-C motif chemokine ligand 5; PGE_2_, Prostaglandin E_2_.

Fluorescence intensity for **Figure 1** was quantified using FIJI (version 2.9.0; available at: https://fiji.sc). Each experiment was independently repeated three times, and ten randomly selected fields were analyzed per replicate. FIJI was also employed to measure the projected areas of spheroids, from which the mean diameter was derived using the formula: $Diameter=2*\sqrt{\frac{Area}{\pi }}$. For the growth curve analyses in **Figures 2**, **3**, experiments were repeated three times, and for each time point, the area and diameter of eight distinct spheroids were calculated. To enhance statistical robustness, experiments for **Figures 4**, **5** were performed in five independent biological replicates, each using cell lines at different passages, and three (for **Figure 4**) to five (for **Figure 5**) different tri-hNSPHs or tri-NSPH-derived supernatants were pooled per condition. Data normality was assessed by integrating the results of the Shapiro–Wilk test with the graphical inspection of Q–Q plots. When data conformed to a normal distribution, statistical significance was determined by one-way analysis of variance (ANOVA), followed by Tukey’s multiple comparison *post hoc* test. For qRT-PCR results showed in **Figure 6**, when the data distribution was not normal, a logarithmic (base 2) transformation was applied; therefore, data are presented as −ΔΔC_t_. Outliers were identified using the ROUT method (Q = 1%) and excluded from analysis, carried out using a two-tailed unpaired t-test with Welch’s correction. Hierarchical clustering and heatmap visualization were performed with ClustVis web tool (https://biit.cs.ut.ee/clustvis/). Gene expression data were row-scaled using Z-score transformation and for sample clustering, correlation was employed as a distance metric. For the analysis of L&D staining experiments (**Figure 3**), fluorescence signals were quantified from 15 distinct tri-hNSPHs, and comparison was performed using a paired two-tailed t-test. Statistical analyses and graph generation were performed using GraphPad Prism (version: 10.5.0; https://www.graphpad.com).

## 3 Results

### 3.1 Differentiation of SH-SY5Y and U138 MG cell lines into neuron-like and astrocyte-like cells

In order to establish a robust culture system resembling primary human neurons, SH-SY5Y cells were differentiated into neuron-like cells (NLCs) with ATRA treatment (24). In parallel, we assessed the contribution of a synthetic extracellular matrix, Geltrex, to neuronal maturation, comparing its effect with that of conventional plastic substrates. Within five days of ATRA exposure, SH-SY5Y cells transitioned from the polygonal morphology to a neuron-like cell phenotype, characterized by spherical soma and elongated axon-like processes forming intricate networks (**Figure 1A**). Consistent with these observations, immunofluorescence staining revealed a robust increase in β3-tubulin expressing cells, paralleled by a reduction of Nestin expressing cells, a marker enriched in progenitor cells (**Figures 1A, B**). Quantitative fluorescence analysis normalized to DAPI signals confirmed these changes, underscoring the acquisition of mature neuron-like identity (**Figures 1C, D**). To complement the phenotypic characterization, we evaluated the transcriptional profile of NLCs. Nurr1, Mash1 and NeuroD1, three transcription factors expressed at the progenitor stage, were markedly downregulated upon differentiation of SH-SY5Y cells into NLCs (**Figure 1E**).

After the establishment of the neuron-like component, we focused on generating astrocyte-like cells (ALCs) suitable for use alongside NLCs in the development of our envisaged 3D model. No significant differences were observed between SH-SY5Y differentiating on plastic or Geltrex, but we selected the second condition for ALCs to keep an environment as similar as possible to a physiological brain, supporting cell maturation with extracellular matrix. We adapted a protocol for glioblastoma differentiation treating U138 MG cell line with sodium butyrate (NaB) for 72 hours. The acquisition of an astrocyte-like phenotype was demonstrated by the increased expression of S100B, a typical marker used for mature astrocytes (**Figures 1F–I**). EdU incorporation assay also revealed a more physiological replication ability, whereby differentiated cells displayed less than half the proliferative activity of the tumorigenic counterpart (**Figures 1G, H**).

### 3.2 Pro-inflammatory stimulation triggers transcriptional profile switch in ALCs and microglia HMC-3 cells

To confirm the responsiveness of the selected cell lines, particularly in view of their incorporation into a 3D model expected to preserve similar properties, ALCs and HMC-3 microglial cells were stimulated for 72 hours with a cytokine cocktail containing IL-1β, TNFα, and IFNγ. Their ability to modulate transcriptional programs was assessed by evaluating a panel of genes associated with the reactive state of each cell type. In ALCs, we quantified the transcriptional levels of well-established pro-inflammatory astrocytic markers, including C3, GBP2, SERPING1, LCN2, and MAFG, as well as the transcription factors STAT3 and NF-κB. In addition, broader inflammatory markers such as GM-CSF, CCL20, and COX2 were examined. As expected, all genes analyzed exhibited a statistically significant increase in expression following cytokine exposure, confirming the ability of ALCs to undergo polarization toward a reactive phenotype (**Figure 2A**). A parallel analysis was performed in HMC-3 cells to evaluate the capacity of this human microglial cell line to elicit a coherent response to pro-inflammatory stimulation. Consistent with the expected activation profile, the pro-inflammatory genes IL-8, MMP3, GM-CSF, IL-12α, NLRP3 and iNOS, as well as the neurotrophin NGF and chemokines CCL20, CXCL1 and CXCL2 displayed a significant upregulation, with the exception of iNOS, which did not reach statistical significance (**Figure 2B**). Heatmap visualization (**Figure 2C**) further highlights a distinct clustering of effector profiles, with ALCs reprogrammed to an activated and neurotoxic phenotype, and HMC-3 cells showing a profile consistent with immune cell recruitment, inflammasome activation and extracellular matrix remodeling.

### 3.3 Development of bipartite hNSPHs (Bi-hNSPHs) and composition assessment

Building on our optimization of protocols for 2D culture and differentiation of SH-SY5Y and U138 MG cells, we next tested the possibility to assemble these cell lines into a more complex, 3D neural model. We started with the development of a bipartite hNSPH model (Bi-hNSPHs) co-culturing SH-SY5Y and U138 MG in methylcellulose to prevent substrate adhesion and to promote spontaneous cellular self-assembly. To ensure uniform nutrient and oxygen distribution, cultures were maintained under dynamic conditions. The 1:1 cell ratio was selected based on the most recent studies on human brain composition that challenged the idea that astrocytes are more abundant than neurons (25, 26). Bi-NSPH growth and morphological development were monitored over 14 days. Bright-field imaging revealed progressive cellular compaction leading to the formation of nearly spherical structures (**Figure 3A**). Spheroid growth proceeded gradually and consistently, with the mean diameter shifting from 400 to 550 µm over time (**Figure 3B**). By day 14, H&E staining of cryosectioned spheroids showed two morphologically distinct cell populations, with a compact cluster of concentrically arranged cells surrounded by a less compact peripheral layer (**Figure 3C**). Immunofluorescence staining for specific markers—β3-tubulin for NLCs (in yellow) and S100β for ALCs (in red)—confirmed that the more cohesive, often eccentrically localized core was predominantly composed of ALCs, whereas the outer, looser cluster consisted mainly of NLCs (**Figure 3D**, upper panel). A deeper characterization of the surrounding neuronal compartment further revealed the presence of Nestin-positive (in green), less mature cells in regions proximal to the astrocytic core, while cells expressing markers of more mature neurons, such as Synapsin (in red) and MAP2 (in magenta), were predominantly localized in the distal areas (**Figure 3D**, lower panels).

### 3.4 Generation and morphological characterization of tripartite hNSPHs

To increase the complexity of our model, aiming for improved similarity with the human brain, we incorporated human microglia-like cells. To this end, we co-seeded the three cell lines at a defined 1:2:4 neuron/astrocyte/microglia ratio into tri-hNSPHs. The culture conditions were the same we optimized for bi-hNSPHs, and the ratio was selected by testing several seeding conditions and selecting the one that ensured stable spheroid formation together with efficient microglial integration *(data not shown)*. We opted for a proportion that yielded a microglial fraction in the range of 5–10%, which is consistent with physiological estimates in the human brain and included comparable numbers of neurons and astrocytes (25–27). Bright-field images revealed progressive compaction and structural refinement (**Figure 4A**). Compared to the bipartite model, which exhibited an irregular and loosely compacted edge, at 14 days tri-hNSPHs displayed a more defined spherical morphology and the outer boundary appeared smoother and uniform (**Figure 4A**). Morphometrically, spheroid diameters expanded from 300–350µm to an average of 550µm by day 14 (**Figure 4B**). For spatial characterization, H&E of tri-hNSPHs identified the same distinct bipartite architecture we saw in bi-hNSPHs, consisting of a dense inner core and a peripheral layer of radially aligned, loosely packed cells, but a markedly higher degree of structural organization, with interconnected neurons and axonal-like projections (**Figure 4C**). At 14 days, cell viability was evaluated with L&D staining. Approximately 94% of the cells in the outer layer of the tri-hNSPHs were calcein-positive (in green), indicating that they were viable and metabolically active, while only about 6% were propidium iodide–positive (in red), corresponding to dead or severely stressed cells (**Figures 4D, E**). A deeper evaluation was not feasible due to instrumental limitations; however, no signs of central necrosis were observed in sliced tri-hNSPHs, highlighting the effectiveness of dynamic culture conditions in supporting viability throughout the spheroid volume.

### 3.5 Tri-hNSPHs contain mature NLCs, ALCs and functioning microglia

To further dissect the microscopic composition of tri-hNSPHs, cryosections collected at different time points were subjected to immunofluorescent analysis using cell type–specific markers: β3-tubulin (in yellow) for NLCs, S100B (in red) for ALCs, and Iba1 (in cyan) for HMC-3 cells. At day 4, the spheroid core was almost entirely populated by ALCs, surrounded by a modest peripheral layer of NLCs. Over time, the neuronal compartment progressively expanded, while the astrocytic core became increasingly compact. Microglial cells were initially localized within the astrocyte-dense core but gradually redistributed, and by day 14, they populated the entire volume of tri-NSHPs (**Figure 5A**). Notably, despite the absence of any exogenous differentiation stimuli, β3-tubulin and S100B expression levels were significantly elevated. This suggested that the co-culture of the three cell types may be sufficient to promote spontaneous cell maturation. This was further corroborated by the expression of other markers associated to mature neurons, Synapsin (in red) and MAP2 (in magenta) (**Figure 5B**). On the contrary, less mature, Nestin-positive cells (in green) were very few and confined near the central cluster of ALCs (**Figure 5B**). In conclusion, to confirm that microglial cells within this context retain their functional properties as antigen presenting cells (APCs), we showed that most HMC3 cells displayed relevant HLA-DR expression (**Figure 5B**).

### 3.6 Tri-hNSPHs is a promising platform to model neuroinflammation and drug screening

To evaluate the potential of tri-hNSPHs for investigating neuroinflammatory processes, spheroids were exposed to a pro-inflammatory cytokine cocktail consisting of IL-1β, TNF-α, and IFN-γ. This stimulation induced a significant inflammatory response, as evidenced by the increase in the endogenous secretion of multiple inflammatory mediators, including IL-6, IL-8, IL-1β, CXCL10, CCL2, CCL5 and PGE_2_. In parallel, we administered 3 compounds—dexamethasone, ibuprofen, and minocycline—to assess their potential in modulating the pro-inflammatory environment within our primed tri-NSPH context. Dexamethasone, a corticosteroid widely used to treat inflammatory disorders, proved to be the most effective compound, markedly reducing the expression of IL-6, IL-8, CCL2, CCL5 and PGE_2_. Minocycline, a tetracycline antibiotic with recognized anti-inflammatory properties, also reduced IL-6, IL-8, CCL5 and PGE_2_ levels, though not those of CCL2. Overall, minocycline effects were less pronounced if compared to the modulation that was induced by dexamethasone. Treatment with the non-steroidal anti-inflammatory drug (NSAID) ibuprofen managed to reduce PGE_2_ but failed to modulate any of the other analyzed cytokines. Rather, it appeared to slightly increase the production of some, such as IL-8 and CCL5, thereby proving ineffective in mitigating the inflammatory activation of tri-hNSPHs. Among all tested compounds, none were able to reduce the secretion of IL-1β and CXCL10 in a statistically significant way (**Figures 6A, B**).

### 3.7 Tri-hNSPHs can be adapted for oxygen deprivation-induced neuropathologies

**Figure 7:**
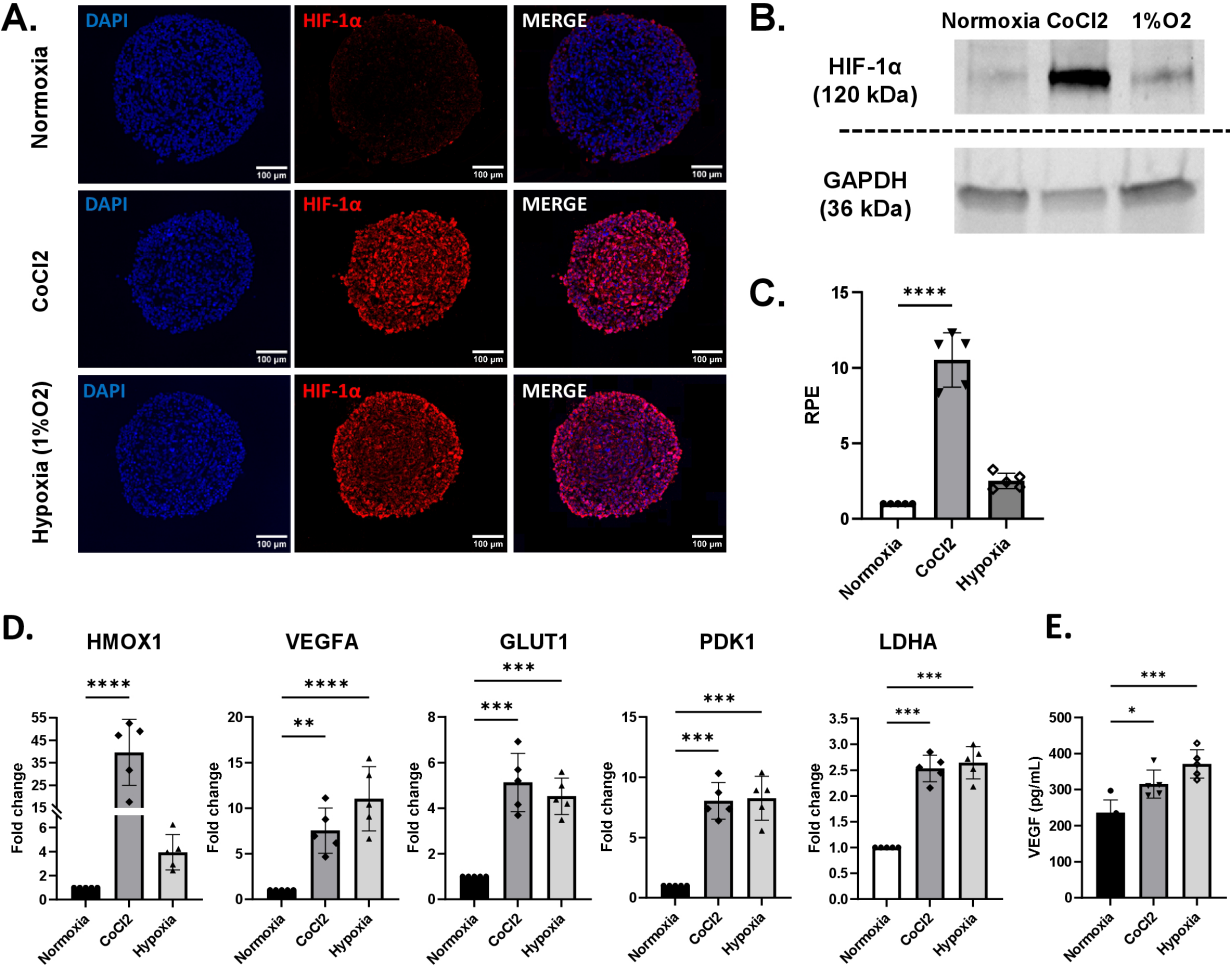
Characterization of the hypoxia-induction treatments in tri-hNSPHs. **(A)** Representative HIF-1α immunofluorescence stainings (in red) of cryosectioned tri-hNSPHs after CoCl_2_ administration and oxygen deprivation. **(B, C)** Quantification of HIF-1α in the nuclear protein fraction of tri-hNSPHs in normoxic and hypoxic conditions, either chemically induced by CoCl_2_ or after cultivation in 1% O_2_ incubator. **(C)** Gene expression analysis of downstream targets of HIF-1α carried out with qRT-PCR. **(D)** VEGF Dosage in supernatants harvested from tri-hNSPHs after 24h of normoxia or hypoxia culture conditions. Data are shown as mean ± SD from 5 independent replicates (n = 5). Statistical significance was determined by one-way ANOVA followed by Tukey’s *post hoc* test. *p< 0.03; **p< 0.02; ***p< 0.0002; ****p< 0.0001. HIF-1α, Hypoxia inducible factor 1 alpha; CoCl2, Cobalt chloride; RPE, Relative protein expression; GAPDH, Glyceraldehyde-3-phosphate dehydrogenase; HMOX1, Heme oxygenase 1; VEGFA, Vascular endothelial growth factor A; GLUT1, Glucose transporter protein type 1; PDK1, Pyruvate dehydrogenase kinase 1; LDHA, L-lactate dehydrogenase A chain.

In addition to mimicking neuroinflammation, we demonstrated that our model is equally suitable for reproducing other pathological contexts that are relevant for the CNS pathophysiology. Specifically, we showed that hypoxia can be successfully induced either by treatment with cobalt chloride (CoCl_2_) or by culturing tri-hNSPHs in an incubator set to 1% O_2_. Immunofluorescent staining showed low levels of HIF-1α either at nuclear and cytosolic localization while a clear stabilization when hypoxia is induced (**Figure 7A**). Moreover, both approaches effectively promoted nuclear translocation of HIF-1α, with CoCl_2_ showing the strongest effect—resulting in more than a tenfold increase in nuclear HIF-1α levels compared with normoxic conditions (**Figures 7B, C**). At the molecular level, hypoxic conditions led to an upregulation of Hmox1, a downstream target of HIF-1α associated with hypoxic and oxidative stress, mirroring the trend of HIF-1α at the protein level. Along with other genes involved in the cellular response to oxygen deprivation, we observed increased expression of VEGFA, a direct transcriptional target of HIF-1α which resulted slightly more up-regulated by the hypoxia induced by the incubator cultivation, as well as GLUT1, PDK1, and LDHA, which collectively indicate a metabolic shift toward anaerobic glycolysis (**Figure 7D**). Finally, as a functional validation of the hypoxic state, quantification of VEGF levels in the tri-NSPH-conditioned medium revealed a statistically significant increase in its secretion if compared with the normoxic culture and the same trend shown for the gene expression (**Figure 7E**).

## 4 Discussion

iPSC-derived brain organoids revolutionized our ability to model the human brain *in vitro*, providing miniaturized systems that mimic its structural and functional complexity with extraordinary fidelity (28, 29). Yet, such sophistication comes at a cost. Their generation demands months of culture, intricate differentiation steps, and significant financial resources, while their inherent batch-to-batch heterogeneity undermines reproducibility (21, 30, 31). Although they represent a milestone in neurobiology, the field increasingly recognizes the need for complementary, simplified systems that retain key physiological features while enabling faster, more affordable, and reproducible experimentation.

In this study, we demonstrated the feasibility of assembling a three-dimensional model of human CNS-like tissue from three well-characterized and widely used cell lines. The resulting model is simple, cost-effective, and highly reproducible, yet it remains well-suited for studying pathophysiological processes affecting the brain. The selected lines have been extensively characterized over the years, with SH-SY5Y cells being a cornerstone of neurobiological research for decades (24). Multiple protocols have been described for inducing the neuronal differentiation of SH-SY5Y cells, ranging from the use of growth factors to simpler, low-cost treatments with ATRA (32, 33). For U138 MG cells, a glioblastoma differentiation protocol that successfully yielded ALCs was adapted (34). Given the extensive literature available on human microglial models, we selected the HMC-3 line as our immune component (35).

The selection of cell lines did not just rely on existing literature; we also generated experimental evidence confirming their ability to mount coherent inflammatory responses. Our analysis focused on ALCs and HMC-3 as astrocytes and microglia are key regulators of neuroinflammation (36, 37). To better mimic conditions relevant to neurodegenerative processes, we employed a sterile inflammatory stimulation (38). IL-1β and TNFα are among the most potent pro-inflammatory cytokines, actively released by microglial cells in response to neural injury. These mediators promote the activation of astrocytes, driving them toward a reactive and neurotoxic phenotype, while further amplifying microglial reactivity (39). IFN-γ, produced by immune cells including microglia, contributes to the disruption of neuronal communication and exacerbates neurodegenerative cascades, notably skewing the microglia phenotype towards M1-like phenotype (40). Upon stimulation, we observed a strong upregulation of the analyzed transcripts (with the exception of iNOS in HMC-3 cells, which did not reach statistical significance), indicating a shift toward a reactive and neurotoxic expression profile (41–43). These results establish a robust 2D inflammatory response framework that supports the development of the 3D model for disease modeling and the study of neuroinflammation.

Bi-hNSPHs, composed solely of SH-SY5Y and U138 MG cells, were our first 3D approach. In a minimal and cost-efficient medium, a compact spherical structure formed within 48 hours and could be maintained for up to 14 days, reaching a size suitable for subsequent analyses. This served as a preliminary step toward our most complete model, in which we successfully integrated microglial cells. This step is particularly challenging, as microglia differ in embryonic origin and are difficult to differentiate simultaneously with neurons and astrocytes (44, 45). In our model, spontaneous integration of microglia occurred without the addition of any exogenous factors, endowing the tri-hNSPHs with intrinsic immunocompetence at no additional cost or complexity. Crucially, the tri-hNSPHs are not a mere aggregation of cells but spontaneously self-organize into a structured cytoarchitecture. Initially, the tri-hNSPHs are dominated by ALCs, but over time, a substantial expansion of the neuronal component is observed. This dynamic reflects the dual mechanical and trophic support that astrocytes provide to neurons. Analysis of neuronal maturation markers revealed that neurons located in the outer layers exhibit a more mature phenotype, expressing markers such as Synapsin and MAP2 (46), whereas Nestin-positive, less differentiated cells (47) were found in proximity to the astrocytic core. This spatial organization effectively recapitulates features of the neurogenic niche, where astrocyte-derived cues sustain neuronal progenitor states (48, 49). Microglial cells also retained their functional identity as evidenced by their sustained expression of HLA-DR, consistent with their antigen-presenting capacity (50). This confirms the microglial component is not merely present, but functionally integrated.

Leveraging this validated, immunocompetent platform, we proved its utility for modeling neuroinflammation. The exposure to the pro-inflammatory mix of cytokines tested on 2D cell cultures induced hallmark features of inflamed neural tissue, most notably the autonomous secretion of inflammatory mediators. This responsiveness, combined with the reproducibility of the system, makes our model particularly appealing as a rapid and scalable drug-screening platform. The translational power of tri-hNSPHs was confirmed by its ability to dissect differential pharmacological responses. Dexamethasone potently suppressed the secretion of most of the cytokines that were dosed, coherently with the well-established glucocorticoid-mediated inhibition of nuclear factor kappa-light-chain-enhancer of activated B cells (NF-κB) and its downstream signaling cascade (51, 52). Notable exceptions were CXCL10 and IL-1β. CXCL10 is an interferon-stimulated gene (ISG) whose transcription is predominantly driven by the Janus kinase/signal transducer and activator of transcription 1 (JAK/STAT1) pathway (53–55). Therefore, inhibition of NF-κB alone may be insufficient to fully abrogate its expression. IL-1β transcription can be modulated by glucocorticoid receptor (56), but its regulation is largely uncoupled from direct corticosteroid control at the level of secretion. Cells typically accumulate IL-1β in its inactive precursor form (pro–IL-1β), which requires post-translational processing, most notably proteolytic cleavage by caspase-1 following NLRP3 inflammasome activation, to generate and secrete the mature cytokine (57, 58). These mechanisms operate independently of classical glucocorticoid receptor-mediated transcriptional repression, providing a plausible explanation for the persistence of IL-1β secretion under dexamethasone treatment. The non-steroidal anti-inflammatory drug ibuprofen showed effect just on PGE_2_, as expected due to its cyclooxygenase (COX) inhibitor activity. Interestingly, the microglial and astrocytic inhibitor minocycline (59, 60) significantly attenuated the inflammatory response, providing direct evidence of the microglial component as an active role in propagating the inflammatory cascade. This capacity to parse distinct cellular contributions and drug mechanisms is a key strength of our platform.

Finally, to further validate the versatility of the model, we demonstrated its applicability to other pathological contexts, such as oxygen deprivation which is a typical situation in ischemic stroke (61). Tri-hNSPHs exposed to either CoCl_2_ or hypoxic culture conditions (1% O_2_) showed coherent activation of the hypoxic response program, including stabilization and nuclear translocation of HIF-1α and induction of its downstream targets. CoCl_2_ chemically induces a condition that resembles hypoxia artificially stabilizing HIF-1α (62), whereas the physical approach recapitulates the hypoxic condition in a more physio-pathological way, with cell reaction provoked by the true absence of oxygen. The difference between the two methods may reflect a stronger alteration of the pathway due to an excessive quantity of uncleared HIF-1a in cells when treated with CoCl_2_. The expression of HMOX1 follows the alteration of HIF-1a, which works as a direct transcription factor (63, 64), but it can be regulated by oxidative stress-induced transcription factors, such as nuclear factor erythroid 2-related factor 2 (Nrf2) (65). Cobalt is toxic and can increase the production of reactive oxygen species (ROS) which, in turn, corroborate the activation of HMOX1 (66, 67). Other canonical downstream targets like VEGFA, GLUT1, PDK1, and LDHA did not show the same magnitude of difference, suggesting a potential saturation effect. The transcriptional machinery at the promoters of these specific glycolytic and angiogenic genes is already maximally occupied/activated by the levels of HIF-1α achieved under physical hypoxia, rendering the “excess” HIF-1α stabilized by CoCl_2_ redundant. The ability to robustly activate this fundamental and distinct pathological pathway, crucial in contexts like stroke and cerebral ischemia (68), underscores the metabolic integrity and adaptability of the model. It confirms the platform is not merely “tuned” to inflammatory stimuli but possesses the functional machinery to study a range of human neuropathologies.

### 4.1 Conclusions and future perspectives

Our model achieved an experimentally relevant level of physiological and pathological complexity, including spatial self-organization and intrinsic immunocompetence, features entirely lacking in 2D cultures. Our tri-hNSPHs bypass key limitations of existing systems, including high production costs and batch-to-batch variability, by offering a simple, affordable, and rapidly generated platform. Despite their robustness and experimental tractability, models based on immortalized cell lines intrinsically carry genetic and epigenetic alterations accumulated during transformation and long-term passaging (69). These abnormalities may distort key signaling and metabolic pathways. The cell lines employed in this study have been rigorously characterized over several decades, becoming extensively-used tools for elucidating mechanisms underlying neurodegeneration (70, 71), neuropsychiatric disorders (72, 73) and neuroinflammation (74, 75). Their large-scale use provides an unparalleled foundation for data comparability, reproducibility, and cross-study validation. The well-established genetic and phenotypic stability constitutes a strength rather than a limitation particularly for hypothesis-driven studies and drug screening applications. Future comparative validation against iPSC-derived organoids and primary cultures will be essential to quantify the degree of similarity between tri-hNSPHs and native human brain tissue. A current limitation of the model is the absence of oligodendrocytes or endothelial cells forming the blood–brain barrier which constrains its ability to fully recapitulate pathological processes such as demyelination and neurovascular responses. Nevertheless, the availability of well-characterized human cell lines representing these missing components provides a foundation for incorporating additional cellular elements to enhance structural and functional fidelity (76, 77). Indeed, this work does not represent an endpoint but rather a starting point toward developing a progressively more complex and physiologically faithful system. Finally, the inherent plasticity of SH-SY5Y cells provides a promising avenue for differentiation into specific neuronal subtypes to create region-specific models of disease (33, 78). Taken together, our findings indicate that this simplified, yet immunocompetent 3D system bridges a critical gap between high−throughput screening and biologically relevant CNS modeling, providing a scalable and valuable tool for preclinical neuroimmunology and therapeutic development.

## Data Availability

The raw data supporting the conclusions of this article will be made available by the authors, without undue reservation.
